# Reciprocal relationships between self-esteem, coping styles and anxiety symptoms among adolescents: between-person and within-person effects

**DOI:** 10.1186/s13034-023-00564-4

**Published:** 2023-02-08

**Authors:** Wenyan Li, Yangfeng Guo, Wenjian Lai, Wanxin Wang, Xiuwen Li, Liwan Zhu, Jingman Shi, Lan Guo, Ciyong Lu

**Affiliations:** 1 Department of Medical Statistics and Epidemiology, School of Public Health, Sun Yat-Sen University, 74 Zhongshan Rd 2, Guangzhou, 510080 China; 2 Health Promotion Centre for Primary and Secondary Schools of Guangzhou Municipality, Guangzhou, 510080 China

**Keywords:** Self-esteem, Coping styles, Anxiety symptoms, Random-intercept cross-lagged panel model, Adolescents

## Abstract

**Background:**

Previous researches have not distinguished the between-person effects from the within-person effects when exploring the relationship between self-esteem, coping styles, and anxiety symptoms among adolescents. To address this gap, this study investigated reciprocal associations among self-esteem, coping styles, and anxiety symptoms in a three-wave longitudinal panel survey, using an analytical strategy that disaggregates the within-person and the between-person variance.

**Methods:**

Data was drawn from the Longitudinal Study of Adolescents’ Mental and Behavioral Well-being Research study conducted in 10 public schools in the Guangdong province of China. All participants had a baseline visit (N = 1957, mean age 13.6, grades 7 and 10) and follow-up interviews at 1-year intervals for 3 years. A random intercept cross-lagged panel model combined with mediation analysis was performed.

**Results:**

At the within-person level, the following results were observed. (1) Low self-esteem and anxiety symptoms bidirectionally predicted each other. (2) Low self-esteem and negative coping style bidirectionally predicted each other. (3) Anxiety symptoms predicted subsequent negative coping style but not vice versa. At the between-person level, we obtained the following main results. (1) Significant predictive effects on the random intercept were found among all three study constructs. (2) There were sex differences regarding the association between self-esteem and anxiety symptoms and the correlation of females was stronger than that of males. (3) Self-esteem mediated the reciprocal relations between coping styles and anxiety symptoms.

**Conclusions:**

Overall, findings revealed a reciprocal relationship between low self-esteem and anxiety symptoms for both females and males. Besides, anxiety symptoms predict subsequent negative coping style but not vice versa. We also highlighted the mediating role of self-esteem in the reciprocal relations between coping styles and anxiety symptoms. Thus, interventions targeted at promoting self-esteem and cultivating positive coping style may help reduce adolescent anxiety.

**Supplementary Information:**

The online version contains supplementary material available at 10.1186/s13034-023-00564-4.

## Background

Anxiety disorders are highly prevalent in children and adolescents, affecting approximately 117 million youth from 5 to 19 years around the world [1]. Anxiety symptoms during adolescence are associated with adverse outcomes across the life course, including anxiety, depression, and substance use persisting into adulthood [2–4]. It not only drastically compromises the daily function of adolescents, but also seriously affects their quality of life [5]. Thus, the identification of modifiable risk factors for anxiety symptoms has significant public health implications [6].

Increasing evidence suggests that self-esteem and coping styles may be risk factors for anxiety symptoms [7–9]. However, the majority of previous studies were mainly based on between-person methodologies to investigate the relationships among self-esteem, coping styles, and anxiety symptoms, such as regression analysis at the group level. Little studies were conducted to investigate whether changes in one variable actually predict another variable at the within-person level. Such inaccurate conclusions conflating between-person and within-person variation, may mischaracterize relationships among self-esteem, coping styles, and anxiety symptoms [10]. Therefore, from a theoretical perspective, the transactional processes within individuals in the associations have not yet been clarified. To address these issues, the random intercepts cross-lagged panel model (RI-CLPM) [10] was employed in this study to test within-person reciprocal relationships between variables (i.e., self-esteem, coping styles, and anxiety symptoms). RI-CLPM combines the advantages of the traditional cross-lagged panel models (CLPM) and overcomes its limitations. RI-CLPM distinguishes within-person effects (i.e., individual-level change, time-varying) from between-person effects (i.e., group-level change, time-invariant) by incorporating random intercepts. This approach contributes to the fundamental understanding of the developmental processes at the within-person level, wherein more accurate causal relationships can be observed [11].

In this study, RI-CPLM was used to determine the direction of within-person relationships between self-esteem, coping styles, and anxiety symptoms as well as the possible mechanisms between the three. Moreover, we further investigated the sex differences in these associations and tested whether these longitudinal associations remained after accounting for common risk factors.

### Self-esteem and anxiety symptoms

Self-esteem is defined as people’s subjective evaluation of their own worthiness as human beings [12]. It has been suggested as an important protective factor for the mental health of adolescents [13]. According to Maslow [7], high self-esteem can positively prompts more prominent happiness and confidence, while low self-esteem possibly leads to inferiority, frustration, hopelessness, and even psychiatric disorders and in particular, anxiety and depression. Terror management theory (TMT) indicates that self-esteem is associated with an increased feeling of safety and security and therefor serves as a buffer against anxiety elicited by awareness of human mortality [14, 15]. A previous cross-sectional study of more than 1000 Norwegian adolescents found that self-esteem was strongly and negatively associated with anxiety and showed a strong protective effect against adolescents’ psychological disease, despite the experience of stressful events [16]. Evidence from longitudinal studies suggested that lower self-esteem predicted prospective elevations of anxiety symptoms [17]. Another recent longitudinal study indicated that low self-esteem could increase the risk of anxiety recurrence after 3 years [13]. To summarize, low self-esteem is considered as a significant risk factor for anxiety symptoms in adolescents.

Despite studies investigating the relationship between self-esteem and anxiety symptoms have identified significant associations, there is little information indicating the reciprocal relations between them and giving inconsistent results. A 2-year longitudinal study of Dutch adolescents (aged 10–16 years) found that low self-esteem at baseline was predictive of relative increases in anxiety symptoms, but baseline anxiety symptoms were not associated with self-esteem at follow-up [18]. However, a meta-analysis reported a possible reciprocal association, where low self-esteem predicts later anxiety symptoms and vice versa [19]. From a theoretical perspective, the causal direction inverse is also plausible. The cognitive model for low self-esteem suggests that prolonged anxiety may have a negative impact on self-confidence, leading to a sense of lower self-esteem and self-worth, and reinforcing negative core beliefs [20]. According to Crocker and Park, experiences of intense anxiety indelibly influence one’s self-concept, thereby persistently threaten and reduce self-esteem [21]. Therefore, we hypothesized that adolescent anxiety symptoms may also undermine self-esteem.

### Self-esteem and coping styles

Adolescence is a transitional period in which individuals strive to search for their own identity and to develop psychosocial competence, including strategies for coping [22]. Coping styles were considered as stable psychological and behavioral strategies that refer to the process of flexibly adjusting cognitive and behavioral strategies to deal with stresses [23]. Coping styles are typically divided into “positive” and “negative”. Positive coping style (i.e., problem-focused coping) refers to an individual’s capacity to cope, adapt, and respond flexibly to adverse circumstances in a positive and rational way. In contrast, negative coping style (i.e., emotion-focused coping) refers to avoidance, social withdrawal, and denial coping process, which could lead to anxiety [24, 25]. What is more, negative coping style may increase the likelihood of negative thoughts and risky behaviors when confronted with stressful events, and positive coping style is protective against some mental disorders, including anxiety, depression, and stress in adolescents [26]. In addition, previous studies reported that self-esteem may contribute to the positive coping style [27, 28].

Empirical studies have provided strong evidence supporting the relationship between self-esteem and coping styles among adolescents. On the one hand, research suggested that high levels of self-esteem as a psychological resource may help enhance individuals’ ability to cope with adversity and enable them to adopt positive coping strategies [27]. Likewise, Michelle et.al has demonstrated that adolescents with high self-esteem can cope with problems better than those with low self-esteem since the former tend to use problem-solving strategies rather than avoidance strategies [28]. Individuals with high self-esteem are more used to evaluating themselves positively and adopting active problem-focused coping strategies, whereas individuals with low self-esteem are more likely to display negative beliefs about themselves and adopt passive emotion-focused coping styles, so they often resort to self-blame, fantasizing, and avoidance [28, 29]. This is a possible mechanism to explain how high self-esteem can function as an important protective factor against long-term psychiatric disorders, such as anxiety [30].

On the other hand, individuals who tend to use positive coping strategies are more likely to keep trying, seek support, and change the value system, so they experience positive emotions more often, including feelings of self-worth and self-confidence [24, 25]. Thus, there may be longitudinal reciprocal relations between self-esteem and coping styles. Heffer et al. using cross-lagged models in a longitudinal study demonstrated that using more positive coping strategies predicted higher self-esteem 1 year later, meanwhile, they also found that lower self-esteem also positively predicted engagement in negative coping strategies 1 year later [31]. However, these bidirectional associations were present in different models, rather than in the same cross-lagged model. Therefore, this prospective bidirectional association between self-esteem and coping styles still needs to be further elucidated.

### Coping styles and anxiety symptoms

The potential association between coping styles and anxiety symptoms was supported in previous studies. Based on the stress-buffering model, engaging in positive coping style can buffer the negative emotion and alleviate psychosomatic symptoms [32]. Several cross-sectional studies among adolescents reported that there was a negative relationship between positive coping style and anxiety symptoms, whereas, the negative coping style was positively related to increased risk for anxiety symptoms [8, 9]. Moreover, Raffety et al. found that higher level of anxiety also lead to negative coping style, such as avoidant coping strategies [33]. However, little researches have evaluated the direct impact of anxiety symptoms on coping styles among adolescents. In one study of adolescents suffering from anxiety and chronic pain, negative coping style played a partial mediating role in the link between anxiety and functional impairment [34]. Further, a recent Australian study evaluated longitudinal associations between coping styles and psychopathology among pre-adolescents in Grade 6, and the results from CLPM showed negative coping style predicted increasing symptoms of generalized and social anxiety, while depressive symptoms, rather than anxiety symptoms, also predicted decreases in positive coping style [35]. Whether there is a bidirectional relationship, and how these relationships change over time is an important issue that remains to be addressed.

Taken together, as discussed above, there might be a bidirectional association among self-esteem, coping styles, and anxiety symptoms, respectively. In addition, there might be mediating effects between the three and the sex-specific differences in these associations might exist [16, 36, 37]. The research model proposed by Julie et al. [38] is based on previous studies showing that individuals with low self-esteem are more likely to use maladaptive rather than adaptive coping, and individuals who use negative coping strategies may be more vulnerable to emotional problems, such as anxiety and depression. However, there is still a paucity of studies addressing the relationships between them and considering both within-person and between-person differences, potential mediating relationships, and possible sex differences simultaneously. Therefore, longitudinal studies were desperately needed to answer these questions.

This study was designed to evaluate the reciprocal within-person relations among self-esteem, coping styles, and anxiety symptoms over time. A three-wave RI-CLPM was employed among Chinese adolescents, making it possible to examine dynamic changes on both individual and intraindividual levels. Drawing upon previous literature, our hypotheses were as follows. (1) Low self-esteem bidirectionally and positively correlated with anxiety symptoms. (2) Low self-esteem bidirectionally and positively correlated with negative coping style. (3) Negative coping style bidirectionally and positively correlated with anxiety symptoms. Moreover, the mediating mechanisms between self-esteem, coping styles, and anxiety symptoms as well as sex differences among them were further investigated; however, no hypotheses were formulated, given the mixed and scarce findings of the existing literature.

## Methods

### Participants and procedure

**Fig. 1:**
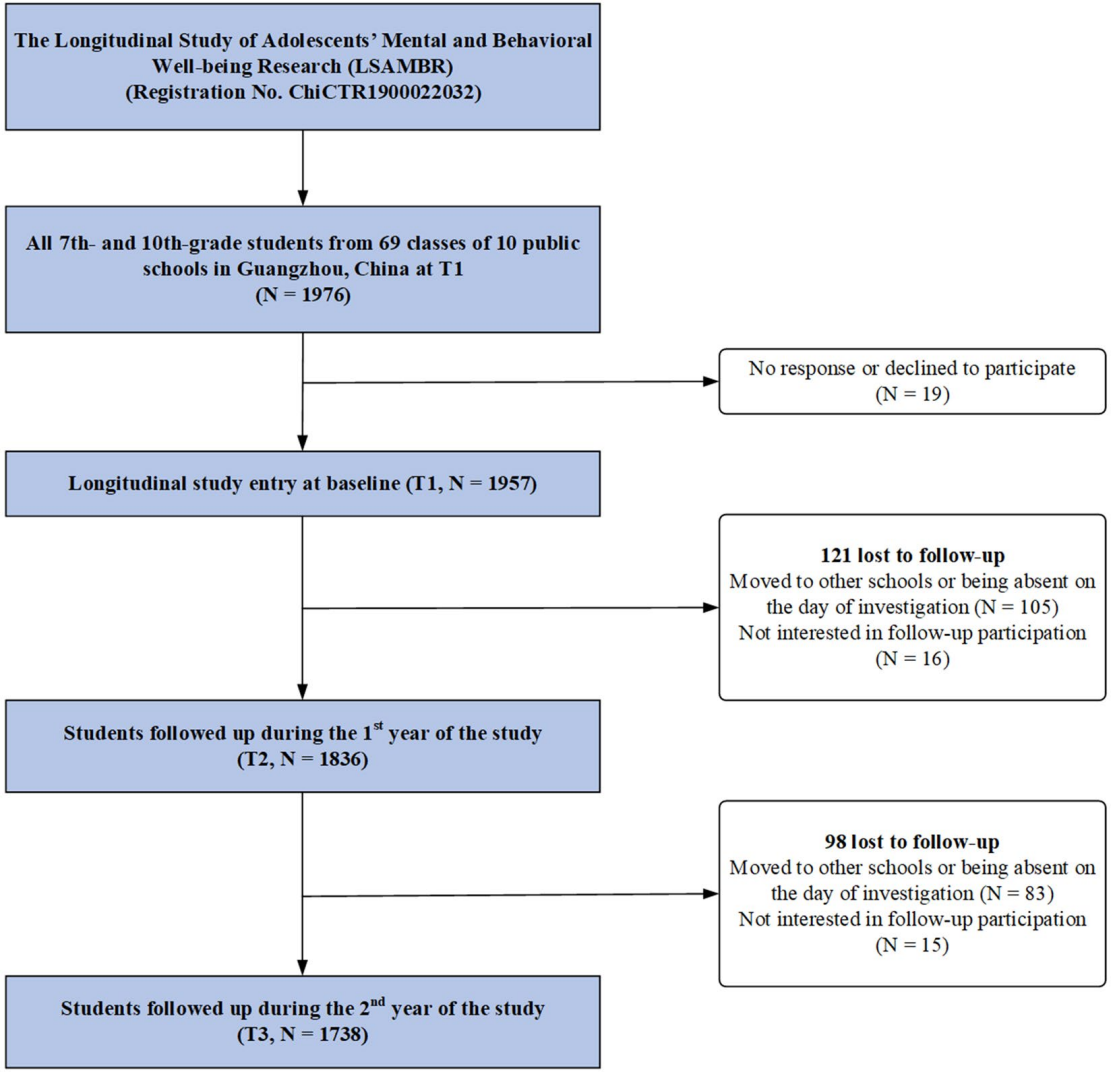
Flow chart of the participants in the present study

The data came from the Longitudinal Study of Adolescents’ Mental and Behavioral Well-being Research (LSAMBR), a 3-year longitudinal investigation (Registration No. ChiCTR1900022032). The LSAMBR recruited representative samples of adolescents from 69 classes of 10 public schools in Guangzhou, China. More detailed information on LSAMBR could be found elsewhere [39]. The current study was based on three waves of the research, each 1 year apart (T1–T3). In total, 1976 students from grades 7 and 10 students were invited at baseline, and 1957 participants were recruited in the cohort between January to April 2019 (T1, response rate: 99.0%). Of those, 1836 completed the 1-year follow-up assessment, 1791 completed the 2-year follow-up assessment, and 1738 (retention rate: 88.8%) completed both follow-up assessments. Finally, this study included 1738 students (Fig. 1). The sample size diminished due to students having transferred out of the school, or being absent at the time of the survey administration. The demographic characteristics (age, sex, household socioeconomic status, living arrangement, and ever drinking) were comparable between the follow-up and lost to follow-up groups (Additional file 1: Table S1).

Students or their legal guardians were informed about the study details and were asked to sign an informed consent prior to the start of the study. Students were excluded if they refused to provide informed consent, were not attending school on the day of survey administration, or refused to take the survey. At each wave, an anonymous, self-administered questionnaire was designed to assess information on socio-demographic characteristics, self-esteem, coping styles, and anxiety symptoms. To avoid information bias, participants were asked to complete the self-administered questionnaires anonymously without the presence of teachers. The study received ethical approval from the Sun Yat-sen University, School of Public Health Institutional Review Board (Ethics Number: L2017060).

### Measures

#### Self-esteem

The Chinese version of the10-item Rosenberg Self-Esteem Scale (RSES) [40] was used to measure general self-esteem. Developed for evaluating global self-esteem in adolescents, this scale included 5 positive items (e.g., “I take a positive attitude toward myself”) and 5 negative items (e.g., “I feel I do not have much to be proud of”), which focus on one’s feelings of respect and acceptance. This scale had been modified according to the characteristics of the Chinese culture [41, 42]. Each item was answered on a 4-point Likert scale ranging from 1 (strongly agree) to 4 (strongly disagree). In its original form, lower scores indicate higher self-esteem. In this study, a reversed rating scale was utilized where a higher score indicated higher level of self-esteem. The Chinese version of this scale has been verified to have good reliability and validity and was wildly used among Chinese adolescents [43]. Cronbach’s alphas in our study were 0.85 (T1), 0.86 (T2), and 0.88 (T3), respectively.

#### Coping styles

The Simplified Coping Style Questionnaire (SCSQ) is a 20-item self-report scale measuring individual coping styles in the context of Chinese culture, which was developed and revised by Xie [25] based on the Ways of Coping Questionnaire (WCQ) by Folkman and Lazarus [44]. It includes 20 items that are divided into two dimensions: positive (12 items) and negative (8 items) coping style. The responses were rated on a 4-point Likert scale (0 = never to 3 = very often). The conversion of the z-score was conducted to calculate standard scores of positive and negative coping style, respectively, according to the average and standard deviation of their original scores. Then, the tendency value of coping styles was calculated by subtracting the standard scores of negative coping from the positive coping style. Hence, the tendency value greater than 0 reflected use of more positive coping style, and less than 0 means adopting a negative coping style [45]. The higher scores indicated a greater tendency to use positive coping style. This scale has presented excellent reliability and validity among Chinese adolescents [39], and the Cronbach’s alphas at T1, T2 and T3 in the current sample were 0.83, 0.85, and 0.86, respectively.

#### Anxiety symptoms

The 7-item Generalized Anxiety Disorder Scale (GAD-7) was used to evaluate the severity of anxiety symptoms over the past 2 weeks. The participants grade the symptoms on a 4-point scale (0 = not at all to 3 = nearly every day) [46]. This scale has good psychometric properties and internal reliability among Chinese adolescents [47]. The global score ranges from 0 to 21, and a higher score implies an increasing severity of anxiety symptoms. In this research, according to the internal consistency test, the Cronbach’s alphas at T1, T2, and T3 in the current sample were 0.89, 0.91, and 0.91, respectively.

#### Covariates

The covariates were investigated at T1, including age, household socioeconomic status, living arrangement, relationships with classmates/teachers, ever smoking, and ever drinking. Household socioeconomic status was assessed by asking: “What is the financial status of your family?” (1 = good, 2 = fair, and 3 = poor). The living arrangements were measured by the question: “Who are you currently living with?” (1 = living with both parents, 2 = living with a single parent, and 3 = living with others). Relationships with classmates/teachers were assessed by the question: “How would you describe your relations with your classmates/teachers?” (1 = good, 2 = average, and 3 = poor). Ever smoking and drinking were measured by the following questions: “Have you ever smoked a whole cigarette?” and “Have you ever drunk beer, wine, or liquor?” (1 = yes, 2 = no).

### Statistical analysis

For descriptive analyses, R version 4.0.3 was used, and for all main analyses, the Mplus version 8.3 was used. Descriptive analyses and bivariate correlations of the relationships between self-esteem, coping styles, and anxiety symptoms were carried out. Next, the Intra-Class Correlation Coefficients (ICCs) were calculated for all study variables. The ICCs were the ratio of the between-person variance to the total variance. The latter is calculated by the sum of between-person variance and within-person variance. Thus, (1 − *ICCs*) indicates the proportion of within-person variance over the measurement waves. If there was substantial within-person variance (i.e., greater than or equal to 10%), the RI-CLPM [10] would be established.

**Fig. 2:**
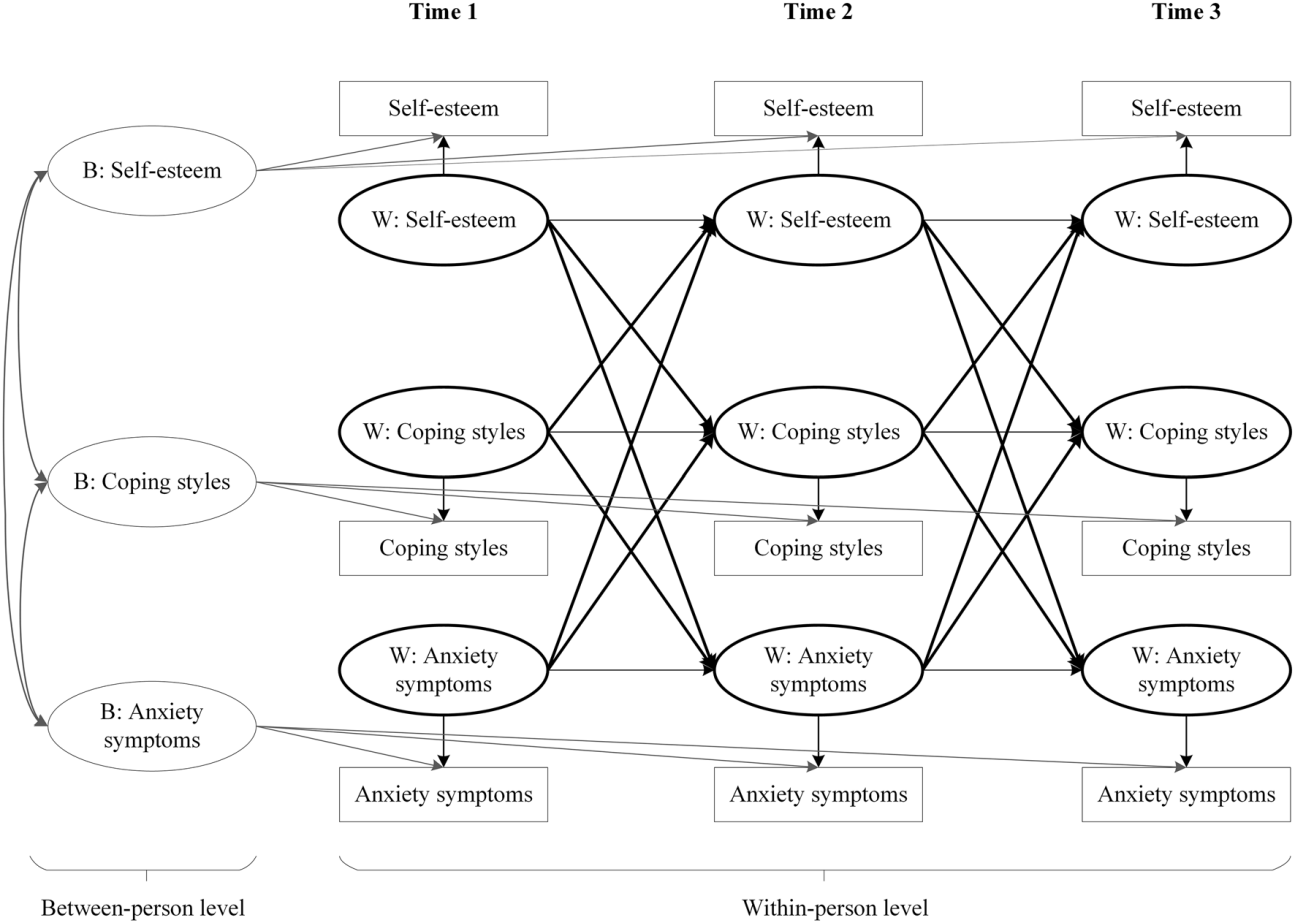
Conceptual random intercepts cross-lagged panel model depicting the relation between self-esteem, coping styles, and anxiety symptoms across three waves. B, between‐person; W, within‐person. In bold are the main associations of interest: between-person level associations are indicated on the left side and within-person level associations are indicated on the right side

The RI-CLPM analysis was employed to examine the directional within-person effects between self-esteem, coping styles, and anxiety symptoms. Compared to CLPM, the RI-CLPM uses a multilevel perspective to divide the variation of each construct (i.e., self-esteem, coping styles, and anxiety symptoms) into two parts [10]. One part is time-invariant between‐person variations in constructs across different time points represented by random intercepts. The second part is the within‐person variations on those same constructs represented by a latent factor for each time point. The random intercepts reflect an individual’s average, so the correlations between the random intercepts represent the between-person effects while the cross-lagged paths represent the time-specific within-person effects. This approach makes the analysis and interpretation more robust and accurate and may provide a foundation for future studies. Figure 2 shows the conceptual model of the RI-CLPM in the present study.

To test our hypotheses, a series of nested models were examined. Firstly, the fit indices of the CLPM (Model 1) and RI-CLPM were compared to determine whether RI-CLPM could accurately fit the data and capture the results. Secondly, to determine the most parsimonious model, the model fits were further compared between the unconstrained models and constrained models in which cross-lagged coefficients and/or auto-regressive paths were restricted to be equal across time. When the models' fits were equally well between the two compared models, the more restrictive model was retained [48]. Thus, four models were compared. Model 2 was an unconstrained model in which all cross-lagged paths, autoregressive paths, and within-time correlations were freely estimated. In Model 3, the within-time correlations and cross-lagged paths of the three variables were constrained to be equal across measurement points. Model 4 was a constrained model in which autoregressive effects were equality constraints. In Model 5, both autoregressive and cross-lagged paths were equally constrained across time points. Thirdly, age, household socioeconomic status, living arrangement, relationships with classmates/teachers, ever smoking, and ever drinking were included in the RI-CLPM as covariates to determine whether the fit of the model was affected by the addition or suppression of covariates (Model 6). Fourthly, to test the sex differences in the hypothesized model, a multiple-group RI-CLPM was conducted. In one model, both paths and covariances were constrained to be equal across sex and compared with another unconstrained model with parameters freely estimated (Model 7). Finally, to test the significance of the mediational paths, we conducted mediation analyses by examining confidence intervals with 1000 bootstraps after selecting the most parsimonious model.

We used a maximum likelihood robust estimator (MLR) to correct for the somewhat skewed distributions and handled missing data with full information maximum likelihood estimation (FIML). For the assessment of the model fit for each model, we evaluated the following indices: chi-square (χ^2^), the comparative fit index (CFI), the Tucker–Lewis fit index (TLI), the root mean square error of approximation (RMSEA) and the standardized root mean squared residual (SRMR). Good model fit was defined as CFI and TLI values > 0.90, RMSEA value < 0.06, and SRMR values < 0.08 [49]. The Satorra–Bentler scaled chi-square difference tests were frequently used to compare model fits [50]. However, the value of chi-square is sensitive to a large sample size, which was not regarded as conclusive [51, 52]. Thus, the ΔCFI and ΔRMSEA greater than 0.01 were used as criteria for determining invariance [51]. The standardized estimates were reported to compare the size of the reciprocal associations at each time point [53]. Full information maximum likelihood was adopted to deal with missing values. All statistical tests were two-sided, and a *p*-value less than 0.05 indicated statistical significance.

## Results

### Descriptive statistics and correlations

The demographic characteristics of the participants at baseline by sex are shown in Additional file 1: Table S2. Among all 1957 participants (mean [SD] age, 13.6 [1.5] years), 994 (50.8%) were males, and 963 (49.2%) were females. The prevalence of living with others, having a poor relationship with teachers, ever smoking, and ever drinking were higher among males than that in females (*p* < 0.05). There were no significant group differences in household socioeconomic status and relationships with classmates.

The correlations among primary study variables at each time point are reported in Table 1. Bivariate correlations demonstrated that significant correlations were found among self-esteem, coping styles, and anxiety symptoms within and across waves (*p* < 0.001). The ICCs were 0.57 for self-esteem, 0.47 for coping styles, and 0.52 for anxiety symptoms. This indicates that 47–57% of the variances in study variables were due to between-person mean differences, and the remaining 43–53% were due to within-person variations. These results suggest sufficient within-person variation to test within-person changes over time by using RI-CLPMs.

**Table 1:** Pearson correlations between self-esteem, coping styles, and anxiety symptoms

Variables	1	2	3	4	5	6	7	8	9
1. Self-esteem T1	1.00								
2. Self-esteem T2	0.63***	1.00							
3. Self-esteem T3	0.50***	0.62***	1.00						
4. Coping styles T1	0.44***	0.34***	0.27***	1.00					
5. Coping styles T2	0.33***	0.44***	0.36***	0.51***	1.00				
6. Coping styles T3	0.31***	0.37***	0.47***	0.42***	0.53***	1.00			
7. Anxiety symptoms T1	− 0.49***	− 0.40***	− 0.31***	− 0.30***	− 0.28***	− 0.23***	1.00		
8. Anxiety symptoms T2	− 0.35***	− 0.48***	− 0.38***	− 0.22***	− 0.34***	− 0.29***	0.54***	1.00	
9. Anxiety symptoms T3	− 0.27***	− 0.36***	− 0.49***	− 0.15***	− 0.26***	− 0.32***	0.44***	0.57***	1.00

Self-esteem was evaluated using the Rosenberg Self-Esteem Scale (RSES); coping styles were evaluated using the Simplified Coping Style Questionnaire (SCSQ); anxiety symptoms were assessed by the Generalized Anxiety Disorder Scale-7 (GAD-7)

T1: time 1; T2: time 2; T3: time 3

***p < 0.001

### Model comparisons

Fit indices for each model and nested model comparisons are presented in Table 2. First, a conventional CLPM was tested (Model 1). However, this model did not provide a good fit to the data according to the fit indices (CFI = 0.964, TLI = 0.867, and RMSEA = 0.090). Therefore, we conducted RI-CLPMs to examine the longitudinal associations among study variables at the within-person level. The fully unconstrained model achieved excellent model fit (Model 2, CFI = 1.000, TLI = 0.995, RSMEA = 0.018, and SMRS = 0.008), and significantly improved the model fit compared with the conventional CLPM (ΔS-B χ^2^ [6] = 140.033, *p* < 0.001; ΔCFI = 0.036, ΔRMSEA = 0.072). Next, we set the contemporaneous as well as cross-lagged paths to be equal across waves, and this nested model (Model 3) did not result in a decline in model fit (CFI = 0.999, TLI = 0.997, RSMEA = 0.014, and SMRS = 0.015), indicating that these path coefficients were equal across waves. Then, the models fixing the auto-regressive paths for self-esteem, coping styles, and anxiety symptoms (Model 4a–c) did not significantly degrade the fit compared with the freely estimated model (*p* > 0.05, ΔCFI < 0.01, ΔRMSEA < 0.01). In the final model, autoregressive and cross-lagged parameters were equally constrained between adjacent waves (Model 5), which had a commensurate fit with the fully unconstrained model (see Table 2, Model 5 vs. Model 2). As presented, the adjusted model (Model 6) still fitted the data well (CFI = 0.989, TLI = 0.982, RMSEA = 0.021) following the inclusion of additional covariates of age, household socioeconomic status, living arrangement, relationships with classmates/teachers, ever smoking, and ever drinking, indicating that the final model construction was reasonable and the fitness was good.

**Table 2:** Model fit indices of cross-lagged structural models and model comparisons for nested models

Models	Fit statistics								Difference tests of relative fit			
	S-B χ^2^	*df*	*p*	CFI	TLI	RSMEA	90% CI	SMRS	Model comparison	DS-B χ^2^ (*df*)^a^	D*p*	Scaling correction factor
1. Conventional cross-lagged panel model with freely estimated paths	152.602	9	< 0.001	0.964	0.867	0.090	0.078–0.103	0.031				1.309
2. Random intercept cross-lagged panel model with freely estimated paths	4.991	3	0.172	1.000	0.995	0.018	0.000–0.046	0.008	2 vs. 1	140.033 (6)	< 0.001	1.156
3. Both contemporaneous and cross-lagged paths are constrained to be equal (= no change over time)	20.696	15	0.147	0.999	0.997	0.014	0.000–0.027	0.015	3 vs. 2	15.737 (12)	0.204	1.192
4 Models with auto-regressive paths are constrained to be equal												
a. Self-esteem paths	4.766	4	0.312	1.000	0.999	0.010	0.000–0.037	0.008	4a vs. 2	0.006 (1)	0.939	1.212
b. Coping styles paths	5.198	4	0.268	1.000	0.998	0.012	0.000–0.038	0.008	4b vs. 2	0.187 (1)	0.665	1.151
c. Anxiety symptoms paths	5.150	4	0.272	1.000	0.998	0.012	0.000–0.038	0.008	4c vs. 2	0.456 (1)	0.500	1.259
5. Select optimal model: reciprocal model with constrained auto-regressive paths and constrained cross-lagged paths	21.960	18	0.234	0.999	0.998	0.011	0.000–0.024	0.016	5 vs. 2	17.061 (15)	0.315	1.228
6. Controlling for covariates based on Model 5	142.532	78	< 0.001	0.989	0.982	0.021	0.015–0.026	0.027				1.115
7. Gender difference												
a. Unconstrained model	71.359	48	0.016	0.994	0.991	0.025	0.011–0.037	0.035				1.245
b. Constrained model	128.921	60	< 0.001	0.983	0.980	0.038	0.029–0.048	0.074	7b vs. 7a	62.900 (12)	< 0.001	1.209

*CFI* comparative fit index, *TLI* Tucker–Lewis Fit Index, *RSMEA* Root Mean Square Error of Approximation, *90% CI* 90% confidence interval of the RSMEA, *D* represents the comparisons of the model fit indexes of S-B χ^2^ and *df*

^a^Chi-square difference calculated using the Satorra–Bentler Scale Chi-square difference test

### RI-CLPM

We tested associations among self-esteem, coping styles, and anxiety symptoms in the RI-CLPM. The constrained RI-CLPM (Model 5) exhibited good fit indices, so this model was therefore used to test the hypotheses. Table 3 shows the standardized coefficients and SE of the hypothesized relationships in RI-CLPM.

**Table 3:** Standardized parameter estimation in the random intercept cross-lagged panel model (RI-CLPM) for self-esteem (SE), coping styles (CS), and anxiety symptoms (AS)

Paths	*β*		SE		95% CI		*p*-value	
Path between random intercepts^a^ (between-person effects)								
SE WITH CS	0.605		0.040		0.526, 0.684		**< 0.001**	
SE WITH AS	− 0.583		0.044		− 0.668, − 0.497		**< 0.001**	
CS WITH AS	− 0.456		0.051		− 0.556, − 0.355		**< 0.001**	

	Time 1 → Time 2				Time 2 → Time 3			
	*β*	SE	95% CI	*p*-value	*β*	SE	95% CI	*p*-value
Autoregressive paths^b^ (within-person effects)								
SE → SE	0.223	0.040	0.144, 0.302	**< 0.001**	0.239	0.046	0.148, 0.329	**< 0.001**
CS → CS	0.146	0.041	0.066, 0.225	**< 0.001**	0.152	0.044	0.065, 0.238	**0.001**
AS → AS	0.217	0.052	0.115, 0.320	**< 0.001**	0.232	0.064	0.108, 0.357	**< 0.001**
Cross-lagged paths^b^ (within-person effects)								
SE → CS	0.073	0.036	0.002, 0.144	**0.044**	0.082	0.041	0.002, 0.162	**0.045**
CS → SE	0.075	0.031	0.015, 0.135	**0.014**	0.074	0.031	0.014, 0.134	**0.015**
SE → AS	− 0.098	0.037	− 0.170, − 0.027	**0.007**	− 0.111	0.040	− 0.189, − 0.033	**0.005**
AS → SE	− 0.125	0.035	− 0.194, − 0.057	**< 0.001**	− 0.127	0.036	− 0.197, − 0.058	**< 0.001**
AS → CS	− 0.082	0.035	− 0.151, − 0.012	**0.021**	− 0.087	0.039	− 0.163, − 0.012	**0.023**
CS → AS	− 0.022	0.032	− 0.085, 0.041	0.501	− 0.023	0.034	− 0.089, 0.044	0.503

Significant paths (*p* < 0.05) are indicated in bold

^a^The values represent the correlation coefficients

^b^The autoregressive and cross-lagged paths were time-invariant. The values represent the standardized regression coefficients

At the between-person level, the random intercepts of self-esteem, coping styles, and anxiety symptoms were significantly related to each other (*p* < 0.001), indicating that low self-esteem was correlated with negative coping styles, and more severe anxiety symptoms, whereas positive coping increased, and the anxiety level decreased further.

After separating the between-person stability, all autoregressive paths were significant (*p* < 0.001). This indicated that within-person variations were significantly predicted by variations at the previous time point (e.g., if individuals reported having higher self-esteem, more positive coping style, and higher anxiety symptoms at T1, they also reported having higher levels of outcomes described above at the subsequent waves). As for the cross-lag effects, there were significant reciprocal within-person associations between self-esteem and coping styles over the three waves. That is, an increase in self-esteem in a given wave was associated with more positive coping style in the next wave (T1–T2: *β* = 0.073, T2–T3: *β* = 0.082, *p* < 0.05). In turn, negative coping style at one-time point was related to decreases in self-esteem at the next time point (T1–T2: *β* = 0.075, T2–T3: *β* = 0.074, *p* < 0.05). Besides, lower self-esteem in a given wave could predict anxiety symptoms in the next wave (T1–T2: *β* = − 0.098, T2–T3: *β* = − 0.111, *p* < 0.01). Conversely, significant negative links from anxiety symptoms in a given wave to self-esteem were demonstrated in the next given wave (T1–T2: *β* = − 0.125, T2–T3: *β* = − 0.127, *p* < 0.001). Moreover, all the paths from coping styles to anxiety symptoms were non-significant, but the paths of inverse association from anxiety symptoms to coping styles showed statistical significance from T1 to T3 (T1–T2: *β* = − 0.082, T2–T3: *β* = − 0.087, *p* < 0.05). This result indicated that bidirectional within-person associations were not present between coping styles and anxiety symptoms.

### Sex differences analyses

To illustrate sex differences, a multi-group RI-CLPM was conducted. In one model, all the paths and covariances were constrained to be equal across sex (i.e., male, female), and the fit of this model was good (model 7b, CFI = 0.983, TLI = 0.980, RSMEA = 0.038 and SMRS = 0.074). Another unconstrained model (model 7a) also exhibited excellent fit indices (CFI = 0.994, TLI = 0.991, RSMEA = 0.025, and SMRS = 0.035). Thus, the unconstrained model showed better fits than the constrained model (ΔS-B χ^2^ [12] = 62.900, *p* < 0.001; ΔCFI = 0.011, ΔRMSEA = 0.013), indicating a significant difference in the paths across sexes. Therefore, the unconstrained model was retained.

**Fig. 3:**
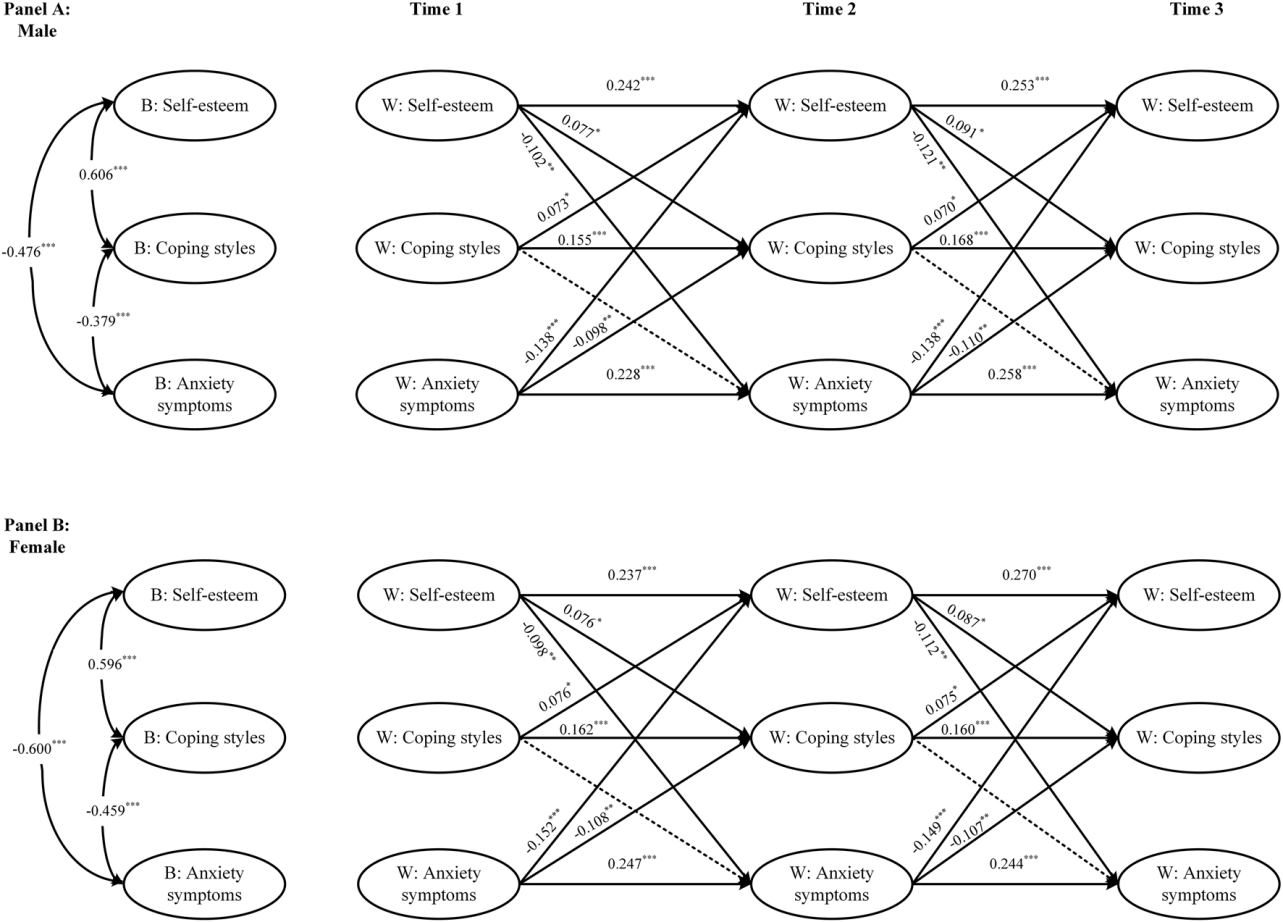
Random intercepts cross-lagged panel model showing relations among self-esteem, coping styles, and anxiety symptoms for males (**A**) and females (**B**) across three waves. Standardized estimates reported. Dashed paths indicated nonsignificant estimates. Indicators, between level intercepts, and within-wave covariances are not shown; estimates are reported in the paper. ***p < 0.001, **p < 0.01, *p < 0.05

The relations among self-esteem, coping styles, and anxiety symptoms for males (Fig. 3A) and females (Fig. 3B) across three waves were presented. Wald chi-square tests showed that, at the between-person level, the association of self-esteem with anxiety symptoms was stronger in females (*r* = − 0.600, *p* < 0.001) than in males (*r* = − 0.476, *p* < 0.001). However, at the within-person level, there was no significant sex difference in the cross-lag paths (Additional file 1: Table S3).

### Mediation analyses

The results of the mediation analyses were depicted in Additional file 1: Table S4. Our results suggested that the paths from coping styles to anxiety symptoms via self-esteem showed statistical significance (*β* = − 0.077, *p* < 0.001), and vice versa (*β* = − 0.006, *p* < 0.001).

## Discussion

In this study, we explored the longitudinal associations among self-esteem, coping styles, and anxiety symptoms by applying RI-CLPM [10] to the three-wave longitudinal study. To our knowledge, this is the first study to comprehensively examine the dynamic effects among these study variables over time in a large sample of Chinese adolescents, which identified both within-person and between-person effects. These results unraveled the precise nature of the reciprocal within-person relations, the mediating mechanisms as well as the sex differences between the aforementioned associations. The findings will contribute to determining potential targets for early identification and intervention that aimed to reduce anxiety symptoms.

### Relations between self-esteem and anxiety symptoms

The results of RI-CLPM indicated that at the between-person level, there was a negative relationship between random intercepts of self-esteem and anxiety symptoms. These findings are supported by previous research suggesting that adolescents who reported lower levels of self-esteem tend to have a higher degree of severity of anxiety [13, 17, 54]. Moreover, the cross-lagged effects at the within-person level were of most interest, as they provided a thorough and rigorous examination that whether study variables predicted one another across waves within persons. In agreement with hypothesis 1, the RI-CLPM analysis demonstrated that self-esteem and anxiety symptoms were bidirectionally associated. An increased level (or a decreased level) of self-esteem resulted in a decreased risk (or an increased risk) for anxiety symptoms in the 1-year follow-up, which in turn can predict an increase (or a decrease) of self-esteem in the 2-year follow-up.

The within-person variations in self-esteem exert a significant negative impact on the within-person variations of anxiety symptoms. Results from the current research further support the previous findings that low self-esteem has a significant relationship with an increased risk for anxiety symptoms in adolescents [13]. Moreover, anxiety symptoms are also the strongest positive predictors of the within-person development of low self-esteem, which is inconsistent with the study conducted by Lonneke et al. They concluded that anxiety symptoms were not a predictor of the future development of low self-esteem [18]. However, our conclusions are in line with a previous meta-analysis, which suggests that self-esteem and anxiety symptoms are bidirectionally related, where low self-esteem predicts later anxiety symptoms and vice versa [19]. These inconsistent results in the previous studies may be due to the study populations, and differences in methodology. Our findings are also consistent with the cognitive model of psychosis [55], which theorizes that low self-esteem is associated with the development of psychotic symptoms. Moreover, experiences of anxiety leave an indelible scar in self-esteem, according to Crocker and Park [21]. Therefore, our findings provide solid evidence for the reciprocal within-person relations between low self-esteem and anxiety symptoms, given the use of RI-CLPM [10].

### Relations between self-esteem and coping styles

At the between-person level, self-esteem is positively associated with coping styles. Adolescents with higher self-esteem are more likely to adopt the positive coping style in general, which corroborates with previous studies [28, 29, 56]. At the within-person level, in agreement with hypothesis 2, the result of RI-CLPM indicated bidirectional within-person associations between self-esteem and coping styles. Individuals with higher self-esteem are more likely to adopt active and positive coping styles than those with lower self-esteem in prior research without examination of within-person changes between the variables over time [28]. Previous results from CLPMs analysis indicate that high self-esteem was a strong predictor of active coping style, whereas the efficiency of coping styles cannot influence self-esteem at any time point [57]. However, our RI-CLPM results reveal reciprocal within-person relations between self-esteem and coping styles after separating the between-person differences. This discrepancy may be due to that the effect of self-esteem on coping styles is predominantly at the within-person level instead of the between-person level. According to the framework for understanding healthy development in the face of risk, high self-esteem and positive coping skills are positive factors that are internal to the individual [58]. As mentioned above, RI-CLPM tests the reciprocal effects within individuals, which controls for between-person differences in self-esteem and coping styles, thus more precise association between these two constructs is observed at the within-person level.

This finding was consistent with the theory presented by Taylor and Brown [59], which supported the hypothesis that people with high levels of self-esteem had more confidence in their abilities, were more likely to adopt positive coping style when facing stressful events, and develop a positive attitude toward life. Not only that, individuals who applied positive coping style to cope with life stressors tended to have better outcomes, which help to the further development of confidence and the improvement of self-esteem [24, 25]. In the future, more longitudinal researches that analyse the variance at both between- and within-person level are warranted to elucidate how self-esteem and coping styles are interrelated over time.

### Relations between coping styles and anxiety symptoms

There was a negative relationship between random intercepts of positive coping style and anxiety symptoms at the between-person level, which was supported by previous studies suggesting that adolescents who used more negative coping styles were more likely to have severe anxiety [8, 9, 35]. Partly in line with hypothesis 3, at the within-person level, adolescents’ anxiety symptoms predicted their subsequent negative coping style but not vice versa. That is, when early adolescents experienced symptoms of anxiety, they are subsequently inclined to take negative coping strategies. On the one hand, our finding is in agreement with the work of Raffety et al., which showed that individuals with negative emotions, such as anxiety or depression, might lead to negative coping style [33]. There is electrophysiological evidence from a human study proved that individuals with more severe anxiety symptoms tended to adopt conservative strategies and pay more attention to negative stimuli [60]. Importantly, there is a good theoretical basis for understanding the role of anxiety as a temporal antecedent in the different coping styles. Major cognitive models of anxiety [61] suggested that the responses to internal (distress) cues and external stimuli would change among individuals with high anxiety, which influence their cognitive processes, social behavior, motivation, and ability to cope with stressful life events. Thus, it is highly plausible that anxiety will predict changes over time in how adolescents cope with stressors. In contrast, a recent longitudinal study using CLPM showed that anxiety symptoms did not predict decreases in positive coping [35]. This discrepancy might due to the methodological difference. In the CLPM, the cross-lagged paths were estimated without unraveling between- and within-person effects, thus these findings may not reflect the actual within-person associations between anxiety symptoms and coping styles over time.

On the other hand, our results reveal that change in coping styles may not lead to another change in anxiety symptoms at the within-person level. This is surprising, given that preliminary indications from the existing longitudinal studies that negative coping predicted increases in symptoms of anxiety [35, 62, 63]. However, none of these studies have disentangled the between- and within-person variations in a single model. Thus, these inconsistencies indicated that the impact of coping styles on anxiety symptoms is mainly at the between-person level (trait-like stability), but not the within-person fluctuations. Moreover, negative coping style may not necessarily exert a direct effect on the anxiety symptoms at the within-person level. For example, Wu et al. proposed that negative coping may elicit anxiety via optimism and psychological capital had a complete mediation effect between positive coping style and anxiety [64].

### The mediating role of self-esteem

While all three constructs, including self-esteem, coping styles, and anxiety symptoms, are important during adolescence and inter-connected, the correlation among them has never been established. In the present study, coping styles did not act as a mediator between self-esteem and anxiety symptoms. However, the RI-CLPMs analysis demonstrated that self-esteem was a significant mediator in the reciprocal associations between coping styles and anxiety symptoms. To be more specific, alterations to coping styles predict the subsequent development of anxiety symptoms via changes in self-esteem and vice versa. Results of our study reveal that coping styles did not exert a direct effect on anxiety symptoms at the within-person level, but had an indirect effect on it through subsequent self-esteem, indicating that adolescents who applied negative coping style tend to report lower self-esteem and increased risk on the development of anxiety symptoms.

These results confirm previous evidence of a positive relationship between negative coping style and low levels of self-esteem [31, 65]. Prior studies showed that psychological capital had a partly or completely mediation effect between coping styles and anxiety [64]. Among psychosocial capital, high self-esteem has been found to unanimously buffer the detrimental effects of stressful life events on psychopathology, acting as a protective factor of anxiety symptoms [13]. A possible explanation might be that adopting positive coping style more often was related to better regulation of behavior and psychology over time, leading to less use of negative coping style and higher self-esteem [31]. Moreover, low self-esteem has been shown to act as a vulnerability factor in the etiology of psychiatric disorders such as anxiety, particularly in adolescents [17]. Inversely, excessive anxiety may cause damage to self-esteem, leaving an indelible scar in the self-concept [21]. Moreover, individuals with low and high self-esteem follow different coping strategies when facing adversities, showing that lower self-esteem was related to more negative coping styles, whereas higher self-esteem was related to more positive coping styles [28]. This could explain the bidirectionality of negative coping styles and anxiety symptoms via low self-esteem.

### Sex differences between self-esteem, coping styles, and anxiety symptoms

In the present study, the RI-CLPM found no sex differences in any cross-lagged effects at the within-person level. That is, the association between the three study constructs was similar between the sexes at the within-person level, which showed the within-person invariance across waves. At the between-person level, we found a significant correlation between the random intercepts of each construct for both males and females, respectively. Interestingly, there was a stronger association between self-esteem and anxiety symptoms among females than in males, demonstrating that females who reported lower levels of self-esteem were more likely to report more severe anxiety symptoms, compared to their male peers. Jong et al. came to a similar conclusion that the relationship between low self-esteem and social anxiety was more evident in girls [36]. Adolescence is the period with substantial change and development in the context of self-esteem as well as psychological health [66]. Previous studies have typically revealed that girls reported higher scores on anxiety [67] and lower scores on self-esteem during adolescence [68]. Self-esteem is the core of self-awareness and an important indicator of mental health [69]. Individuals with low self-esteem usually have a higher risk for mental illness and a high incidence of anxiety symptoms [70]. Hence, girl adolescents may be an especially vulnerable population of mental health difficulties that requires particular attention.

### Strengths and limitations

There were several major strengths of the present study. First, a longitudinal design using RI-CLPM was adopted to identify more precise predictive relations by accounting for time sequence as well as stability effects simultaneously. In this study, we explored how self-esteem, coping styles, and anxiety symptoms concurrently and longitudinally influenced each other over time at the within-person level. Compared with previous researches, our study design and methodology shed more light on this issue. Second, the RI-CLPMs employed in the present study differentiated within‐person from between‐person variance, which makes the results of this study more stable and reliable for within-person bidirectional relations. It will ultimately provide important practical implications for psychiatrists and policymakers working with adolescents. Specifically, between-person results determined which populations needed active intervention, and then within-person results shed light on the mechanisms by which self-esteem and coping styles contributed to anxiety, so as to provide better targets for intervention. Third, few studies have explored the direction of causal relationships among self-esteem, coping styles, and anxiety symptoms across waves among adolescents within the Asian cultural context. This study extends previous work in a unique Chinese context that finds more distributed mechanisms of stable personal characteristics (e.g., self-esteem and coping styles) on anxiety, thus making a significant complement to existing literature.

This study also presented with several limitations. First, due to data collection limitations, the status of anxiety symptoms was based on the respondents’ self-reports rather than clinical diagnoses, which though validated and widely used, may lead to a reporting bias. Second, all of our participants came from one city in southern China, which restricted the generalization of the results. Therefore, research that replicates our findings in different areas and cultural contexts would be meaningful to further validate our results. Third, though we have controlled for several important covariates, some unmeasured covariates (e.g., genetic factors and negative life events) [71] have not been adjusted, which may have an impact on the present outcomes.

## Conclusions

This study contributes to addressing methodological challenges in previous literature by investigating the associations among self-esteem, coping styles, and anxiety symptoms across three waves using RI-CLPM. Overall, considering the between-person effects, both high self-esteem and positive coping style may have a significant negative effect on the random intercept of anxiety symptoms. At a within-person level, findings support that low self-esteem predicts anxiety symptoms and vice versa. Sex differences are found in this study, wherein the association between self-esteem and anxiety symptoms is stronger among females than in males. Moreover, anxiety symptoms predict subsequent negative coping style but not vice versa at a within-person level. Mediation analysis results indicate that the prospective associations between coping styles and anxiety symptoms are mediated by self-esteem. Therefore, adolescents who display a low level of self-esteem are in special need of attention, especially females. Prevention and intervention promoting the level of self-esteem and cultivating the positive coping style are likely to contribute to the reduction of anxiety symptoms.

## Supplementary Information

**Additional file 1: Table S1.** Baseline sample characteristics between follow-up and loss to follow-up participants. **Table S2.** The demographic characteristics of the study participants at baseline by sex. **Table S3.** Chi-square statistics for sex differences in specific paths of the selected models. **Table S4.** Significant indirect paths between self-esteem, coping styles, and anxiety symptoms for the RI-CLPM (Standardized Coefficients).

## Data Availability

The datasets used and/or analysed during the current study are available from the corresponding author on reasonable request.

## Abbreviations

RI-CLPM: Random intercepts cross-lagged panel model
CLPM: Cross-lagged panel models
TMT: Terror management theory
LSAMBR: Longitudinal Study of Adolescents’ Mental and Behavioral Well-being Research
RSES: Rosenberg Self-Esteem Scale
SCSQ: Simplified Coping Style Questionnaire
WCQ: Ways of Coping Questionnaire
GAD-7: The 7-item Generalized Anxiety Disorder Scale
ICCs: Intra-Class Correlation Coefficients
MLR: Maximum likelihood robust estimator
FIML: Full information maximum likelihood estimation
χ^2^: Chi-square
CFI: Comparative fit index
TLI: Tucker–Lewis fit index
RMSEA: Root mean square error of approximation
SRMR: Standardized root mean squared residual
