# Supplementary material for: Reciprocal relationships between self-esteem, coping styles and anxiety symptoms among adolescents: between-person and within-person effects
Source: Child Adolesc Psychiatry Ment Health. 2023 Feb 8;17:21. doi: 10.1186/s13034-023-00564-4 (PMC9909938; doi:10.1186/s13034-023-00564-4)
Supplement: Supplementary file 1 — Additional file 1: Table S1. Baseline sample characteristics between follow-up and loss to follow-up participants. Table S2. The demographic characteristics of the study participants at baseline by sex. Table S3. Chi-square statistics for sex differences in specific paths of the selected models. Table S4. Significant indirect paths between self-esteem, coping styles, and anxiety symptoms for the RI-CLPM (Standardized Coefficients). [file 13034_2023_564_MOESM1_ESM.docx]

Additional file 1

| **Table S1.** Baseline sample characteristics between follow-up and loss to follow-up participants. | | | |
| --- | --- | --- | --- |
| Variables | Follow-up | Loss to follow-up | *p^*^* |
|  | (*n* = 1738) | (*n* = 219) |  |
| Age (year), mean ± SD | 13.6 ± 1.5 | 13.5 ± 1.5 | 0.923 |
| Sex |  |  |  |
| Male | 875 (50.3) | 119 (54.3) | 0.297 |
| Female | 863 (49.7) | 100 (45.7) |  |
| HSS |  |  | 0.127 |
| Good | 893 (51.5) | 119 (54.6) |  |
| Fair | 776 (44.8) | 86 (39.4) |  |
| Poor | 64 (3.7) | 13 (6.0) |  |
| Missing data | 5 | 1 |  |
| Living arrangement |  |  | 0.066 |
| Living with both parents | 1422 (82.0) | 172 (78.5) |  |
| Living with a single parent | 161 (9.3) | 31 (14.2) |  |
| Living with others | 151 (8.7) | 16 (7.3) |  |
| Missing data | 4 | 0 |  |
| Classmate relations |  |  | 0.027 |
| Good | 1488 (85.8) | 175 (80.6) |  |
| Average | 220 (12.7) | 34 (15.7) |  |
| Poor | 26 (1.5) | 8 (3.7) |  |
| Missing data | 4 | 2 |  |
| Relationship with teachers |  |  | 0.005 |
| Good | 1438 (83.6) | 167 (76.3) |  |
| Average | 262 (15.2) | 45 (20.5) |  |
| Poor | 20 (1.2) | 7 (3.2) |  |
| Missing data | 18 | 0 |  |
| Ever smoking a cigarette |  |  | 0.009 |
| Yes | 20 (1.2) | 8 (3.7) |  |
| No | 1708 (98.8) | 211 (96.3) |  |
| Missing data | 10 | 0 |  |
| Ever drinking alcohol |  |  | 0.079 |
| Yes | 556 (32.2) | 84 (38.4) |  |
| No | 1172 (67.8) | 135 (61.6) |  |
| Missing data | 10 | 0 |  |
| Note: Data are given as number (percentage) unless otherwise indicated.  *The chi-square tests were used for categorical variables, and the *t*-tests were used for age. | | | |

| **Table S2.** The demographic characteristics of the study participants at baseline by sex. | | | | |
| --- | --- | --- | --- | --- |
| Variables | Total sample | Males | Females | *p^*^* |
|  | (*n* = 1957) | (*n* = 994) | (*n* = 963) |  |
| Age (year), mean ± SD | 13.6 ± 1.5 | 13.6 ± 1.5 | 13.5 ± 1.4 | 0.032 |
| HSS |  |  |  | 0.107 |
| Good | 1012 (51.9) | 495 (50.0) | 517 (53.8) |  |
| Fair | 862 (44.2) | 449 (45.4) | 413 (43.0) |  |
| Poor | 77 (3.9) | 46 (4.6) | 31 (3.2) |  |
| Missing data | 6 | NA | NA |  |
| Living arrangement |  |  |  | 0.047 |
| Living with both parents | 1594 (81.6) | 805 (81.2) | 789 (82.0) |  |
| Living with a single parent | 192 (9.8) | 88 (8.9) | 104 (10.8) |  |
| Living with others | 167 (8.6) | 98 (9.9) | 69 (7.2) |  |
| Missing data | 4 | NA | NA |  |
| Classmate relations |  |  |  | 0.522 |
| Good | 1663 (85.2) | 838 (84.6) | 825 (85.9) |  |
| Average | 254 (13.0) | 137 (13.8) | 117 (12.2) |  |
| Poor | 34 (1.7) | 16 (1.6) | 18 (1.9) |  |
| Missing data | 6 | NA | NA |  |
| Relationship with teachers |  |  |  | 0.002 |
| Good | 1605 (82.8) | 792 (80.4) | 813 (85.2) |  |
| Average | 307 (15.8) | 172 (17.5) | 135 (14.2) |  |
| Poor | 27 (1.4) | 21 (2.1) | 6 (0.6) |  |
| Missing data | 18 | NA | NA |  |
| Ever smoking a cigarette |  |  |  | 0.017 |
| Yes | 28 (1.4) | 21 (2.1) | 7 (0.7) |  |
| No | 1919 (98.6) | 969 (97.9) | 950 (99.3) |  |
| Missing data | 10 | NA | NA |  |
| Ever drinking alcohol |  |  |  | 0.007 |
| Yes | 640 (32.9) | 353 (35.7) | 287 (29.9) |  |
| No | 1307 (67.1) | 635 (64.3) | 672 (70.1) |  |
| Missing data | 10 | NA | NA |  |
| Time 1 |  |  |  |  |
| Self-esteem, mean ± SD | 28.79 ± 4.80 | 29.30 ± 4.61 | 28.26 ± 4.93 | <0.001 |
| Positive coping style, mean ± SD | 18.76 ± 7.62 | 18.31 ± 8.00 | 19.21 ± 7.19 | 0.010 |
| Negative coping style, mean ± SD | 7.33 ± 4.42 | 6.99 ± 4.46 | 7.68 ± 4.35 | 0.001 |
| Anxiety symptoms, mean ± SD | 3.76 ± 4.30 | 3.19 ± 3.91 | 4.35 ± 4.60 | <0.001 |
| Time 2 |  |  |  |  |
| Self-esteem, mean ± SD | 28.94 ± 4.97 | 29.41 ± 4.91 | 28.47 ± 4.99 | <0.001 |
| Positive coping style, mean ± SD | 18.81 ± 7.93 | 18.49 ± 8.35 | 19.12 ± 7.47 | 0.093 |
| Negative coping style, mean ± SD | 7.28 ± 4.55 | 6.97 ± 4.68 | 7.59 ± 4.40 | 0.004 |
| Anxiety symptoms, mean ± SD | 3.60 ± 4.31 | 3.02 ± 4.16 | 4.18 ± 4.38 | <0.001 |
| Time 3 |  |  |  |  |
| Self-esteem, mean ± SD | 28.96 ± 5.00 | 29.20 ± 4.95 | 28.72 ± 5.05 | 0.040 |
| Positive coping style, mean ± SD | 18.46 ± 7.84 | 18.01 ± 8.19 | 18.91 ± 7.45 | 0.017 |
| Negative coping style, mean ± SD | 7.35 ± 4.67 | 7.07 ± 4.76 | 7.64 ± 4.56 | 0.010 |
| Anxiety symptoms, mean ± SD | 3.57 ± 4.18 | 2.97 ± 3.93 | 4.18 ± 4.34 | <0.001 |
| Note: Self-esteem was evaluated using the Rosenberg Self-Esteem Scale (RSES); coping styles were evaluated using the Simplified Coping Style Questionnaire (SCSQ); anxiety symptoms were assessed by the Generalized Anxiety Disorder Scale-7 (GAD-7). NA, not applicable or no data available. Data are given as number (percentage) unless otherwise indicated. *The chi-square test was used for categorical variables, and the *t*-test was used for age, self-esteem, coping styles, and anxiety symptoms. | | | | |

| **Table S3.** Chi-square statistics for sex differences in specific paths of the selected models. | | | |
| --- | --- | --- | --- |
|  | Wald χ^2^ | *df* | *p* |
| 1. Reciprocal paths |  |  |  |
| a. CS → SE | 0.213 | 1 | 0.644 |
| b. AS → SE | 0.290 | 1 | 0.590 |
| c. SE → CS | 1.061 | 1 | 0.303 |
| d. AS → CS | 2.139 | 1 | 0.144 |
| e. SE → AS | 1.924 | 1 | 0.165 |
| f. CS → AS | 1.128 | 1 | 0.288 |
| 2. Auto-regressive paths |  |  |  |
| a. SE → SE | 0.946 | 1 | 0.331 |
| b. CS → CS | 0.298 | 1 | 0.585 |
| c. AS → AS | 2.253 | 1 | 0.133 |
| 3. Between-person correlation |  |  |  |
| a. SE WITH CS | 1.138 | 1 | 0.286 |
| b. SE WITH AS | 6.636 | 1 | 0.010 |
| c. CS WITH AS | 3.441 | 1 | 0.064 |
| SE: self-esteem; CS: coping styles; AS: anxiety symptoms.  The results for the Wald Chi-square tests are presented. | | | |

| **Table S4.** Significant indirect paths between self-esteem, coping styles, and anxiety symptoms for the RI-CLPM (Standardized Coefficients). | | | | |
| --- | --- | --- | --- | --- |
| **Indirect Effects** | **RI-CLPM** | | | |
|  | ***β*** | **Standard error** | **Bootstrapped 95% CI** | |
|  |  |  | **Lower** | **Upper** |
| SE T1 → AS T2 → CS T3 | 0.002 | 0.001 | 0.000 | 0.004 |
| **CS T1 → SE T2 → AS T3** | **-0.077***** | 0.019 | -0.115 | -0.040 |
| **AS T1 → SE T2 → CS T3** | **-0.006***** | 0.002 | -0.009 | -0.003 |
| AS T1 → CS T2 → SE T3 | -0.005 | 0.003 | -0.012 | 0.001 |
| SE: self-esteem; CS: coping styles; AS: anxiety symptoms; T: Time; RI-CLPM: Random Intercept Cross-Lagged Panel Model; CI: confidence interval.  Significant paths (*p* < 0.05) are indicated in bold.  ****p* < 0.001 | | | | |
